# Synonymous nucleotide changes drive papillomavirus evolution

**DOI:** 10.1016/j.tvr.2022.200248

**Published:** 2022-10-17

**Authors:** Kelly M. King, Esha Vikram Rajadhyaksha, Isabelle G. Tobey, Koenraad Van Doorslaer

**Affiliations:** a School of Animal and Comparative Biomedical Sciences, University of Arizona, Tucson, AZ, USA; b Department of Physiology and Department of Ecology and Evolutionary Biology, University of Arizona, Tucson, AZ, USA; c Cancer Biology Graduate Interdisciplinary Program, University of Arizona, Tucson, AZ, USA; d The BIO5 Institute, The Department of Immunobiology, Genetics Graduate Interdisciplinary Program, UA Cancer Center, University of Arizona Tucson, Arizona, USA

## Abstract

Papillomaviruses have been evolving alongside their hosts for at least 450 million years. This review will discuss some of the insights gained into the evolution of this diverse family of viruses. Papillomavirus evolution is constrained by pervasive purifying selection to maximize viral fitness. Yet these viruses need to adapt to changes in their environment, e.g., the host immune system. It has long been known that these viruses evolved a codon usage that doesn't match the infected host. Here we discuss how papillomavirus genomes evolve by acquiring synonymous changes that allow the virus to avoid detection by the host innate immune system without changing the encoded proteins and associated fitness loss. We discuss the implications of studying viral evolution, lifecycle, and cancer progression.

## 1 The family *Papillomaviridae*

**Fig. 1:**
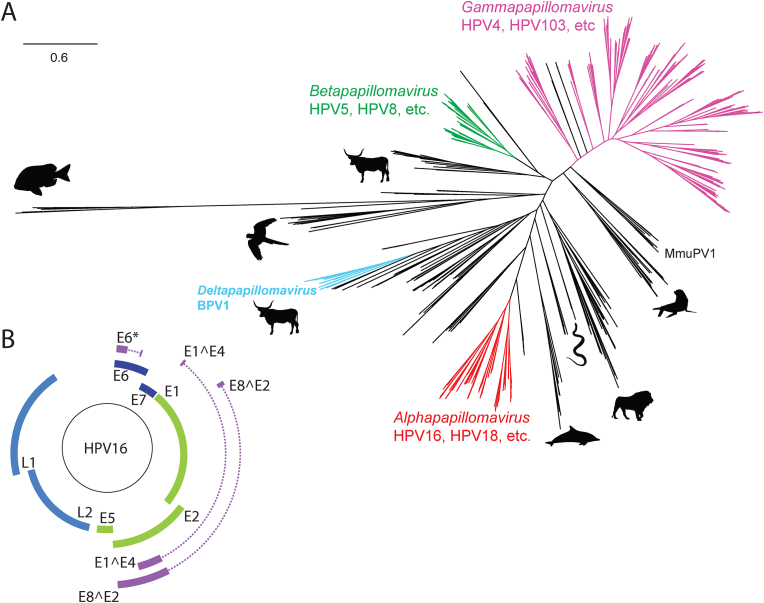
(A) Papillomavirus phylogenetic tree. The protein sequence coding for E1, E2, and L1 for all 667 papillomaviruses currently on PaVE ([1,2]; June 2022) were downloaded and aligned. A concatenated phylogeny was constructed using default parameters for FastTree. Members of the genera *Alphapapillomaviurs, Betapapillomavirus, Gammapapillomavirus,* and *Deltapapillomavirus* are indicated [[3], [4], [5]]. The different animal silhouettes provide a broad overview of animal tropism. (B) Diagram of a typical human papillomavirus genome belonging to the genus *Alphapapillomavirus*. The outer boxes indicate the protein-coding open reading frames. Dotted lines represent intron sequences. Diagram was downloaded from PaVE and edited in Adobe Illustrator.

The family *Papillomaviridae* contains small, non-enveloped viruses with double-stranded genomes ranging between 5748 bp (Sparus aurata papillomavirus type 1; SaPV1) to 8607 bp (canine papillomavirus type 1; CPV1). Papillomaviruses have been isolated from fish, reptiles, birds, and mammals. As the most sampled host, 441 unique papillomaviruses have been isolated from humans. The Papillomavirus Episteme (PaVE; [1,2]) contains 664 genetically distinct papillomavirus types, classified into two subfamilies, >50 genera and >130 species [[1], [2], [3]] (Fig. 1A). Classification of papillomaviruses is based on pairwise nucleotide sequence identity across the L1 open reading frame [3]. Distinct papillomavirus types share less than 90% sequence identity across the L1 open reading frame [3]. The original criteria distinguishing genera stated: “*Most types within a PV genus show less than 60% sequence identity to types of other genera based on global multiple sequence or pairwise alignments of the L1 genes. Nevertheless, the suggested percentage identities that define PV genera have to be taken as general, but not absolute criteria as curation is necessary*” [4,5]. Practically, papillomavirus genera and species are primarily delineated by visual inspection of phylogenetic trees derived from concatenated E1, E2, and L1 nucleotide sequences [3]. Efforts are underway to refine the papillomavirus classification scheme and update viral species names in line with recent changes by the International Committee for the Taxonomy of Viruses (ICTV) [6].

## 2 The viral lifecycle

Members of the *Papillomaviridae* family primarily infect mucosal and keratinized epithelia. Papillomavirus virions are non-enveloped with a capsid made up of 360 copies (arranged as 72 pentamers) of the major capsid protein, L1, and up to 72 molecules of the L2 minor capsid protein [7]. Each capsid packages a single copy of the viral circular dsDNA associated with core histone proteins [8,9].

Temporal expression of the viral genome is associated with tissue differentiation and is tightly regulated at the level of transcription and RNA processing [[10], [11], [12]]. A typical mammalian papillomavirus encodes six to nine proteins (Fig. 1B). However, only 4 proteins (E1, E2, L1, and L2) can be identified in all papillomaviruses sequenced to date [13,14]. The viral helicase, E1, is essential for the replication and amplification of the viral chromosome in the nucleus of infected cells [15]. The E2 protein regulates viral transcription, initiation of DNA replication, and partitioning of the viral genome [16]. The additional viral proteins likely play essential yet supporting roles in the viral lifecycle.

The E6 and E7 proteins are critical in creating a cellular milieu that supports the viral lifecycle [17,18]. Specifically, since papillomaviruses replicate in differentiating tissues, E6 and E7 uncouple viral replication from cellular differentiation. In a subset of papillomaviruses, the E6 and E7 proteins are the primary oncogenes [19,20]. The E1^E4 gene product is typically translated from a spliced mRNA fusing approximately the first four E1 codons to the E4 ORF, present in an alternative reading frame to the E2 ORF. The role of the E4 protein is not entirely clear, but E4 serves as a biomarker of active virus infection [21]. E8^E2 is a similarly alternatively spliced gene product. The E8 exon is embedded within E1 and utilizes the same splice acceptor site as E1^E4 mRNA, generating mRNA for the E8^E2 protein. E8^E2 inhibits viral replication and gene expression [22]. These alternatively spliced genes appear to be unique to papillomaviruses that infect mammals. A subset of viral mRNA encodes a short, hydrophobic, transmembrane protein, E5 or E10. E5 proteins are typically encoded in the 3ʹ-end of the early coding region [23,24]. The E10 proteins are located in this region without an E6 gene [14]. Most of these ‘early genes’ are transcribed as polycistronic mRNAs using the same promoter and identical poly-A termination signal.

Interestingly, the E8^E2 gene product is derived from a unique promoter in E1, suggesting that E8^E2 can be separately regulated throughout the viral lifecycle. As the infected cell differentiates, the viral genome is amplified, and the viral capsid proteins, L1 and L2, are expressed. The major capsid protein L1 contributes to the main structural component of the viral capsid [25]. In contrast, the minor capsid protein L2 is critical for viral assembly and subsequent infection of a new host cell [[26], [27], [28]].

## 3 Host and virus evolution are linked, but it is complicated

Most papillomavirus infections cause benign, mostly unapparent, persistent infections in their hosts. In addition, papillomaviruses are highly host-restricted and cause abortive infections in non-host species [29,30]. The observation that papillomaviruses cause highly host-restricted and primarily benign infections led to the hypothesis of virus-host co-speciation or “host-linked evolution” [13,31].

Traditionally, co-speciation theory implies that the phylogenetic tree of the viruses and the phylogenetic tree of infected hosts should closely mirror each other (Fahrenholz's rule; [32]).

However, with the increasing number of viruses being identified in different animal hosts, it is clear that strict co-speciation between virus and host is not supported by the data [24,30,[33], [34], [35], [36], [37], [38], [39], [40], [41], [42], [43], [44]]. Indeed, it seems unlikely that a single ancestral papillomavirus infected an ancestral host and both virus and host co-speciated, generating the extant diversity in papillomavirus genome sequences. Several violations of a strict-virus-host co-speciation can be seen in the phylogenetic tree in Fig. 1A. A similar but interactive and zoomable phylogenetic tree can be accessed through the PaVE website (https://pave.niaid.nih.gov/analyze/phylogenetic_tree; accessed June 2022). For example, human papillomaviruses can be found in five different genera (*Alphapapillomavirus, Betapapillomavirus, Gammapapillomavirus, Mupapillomavirus,* and *Nupapillomavirus*) in different locations throughout the phylogenetic tree. Under strict co-speciation, all human viruses would be expected to cluster together in a single clade.

Another striking example is two papillomaviruses isolated from snakes. BcosPV1 [43] and MsPV1 [44] were isolated from a Boa constrictor and Diamond Python, respectively. Based on co-speciation and the location of other viruses isolated from reptile hosts, we expect these viruses to cluster near the root of the phylogenetic tree (parrot silhouette in Fig. 1A). However, these snake viruses cluster among mammalian viruses (snake silhouette).

The observed discrepancy between the host and virus trees has been explained by evolutionary events such as horizontal gene transfer, recombination, and virus duplication (e.g., following ecological niche adaptation) [30,[45], [46], [47], [48], [49], [50]]. While recombination likely played a role at specific moments throughout viral evolution, its overall importance during viral evolution is likely limited (reviewed in Ref. [13]). Since papillomaviruses are highly species-specific, horizontal gene transfer likely did not play any role in the evolution of the *Papillomaviridae* [48]. It is important to point out that more complete and unbiased sequencing of animal papillomavirus diversity will likely help to clarify the evolutionary history of the *Papillomaviridae*.

An updated version of the co-evolution theory suggests that viruses follow the evolution of host resources [51]. Under this model, a generalist ancestral virus was able to thrive in multiple tissue niches. During the late Devonian, some infected ancestral animal adapted to life on land. Specific events in the evolution of these ancestral hosts (e.g., the presence/absence of fur, the evolution of sweat glands, etc.) created new ecological niches for papillomaviruses to adapt [31]. Resource-driven evolution would have driven niche adaptation allowing for specialization and virus duplication. This process would explain the diversification of papillomaviruses into four or five groups of increasingly specialized viruses (reflected in the 4–5 major clades of the phylogenetic tree; Fig. 1A) [33]. Following these niche adaptation events, virus-host co-speciation was the main driver of virus diversification. Throughout the co-speciationary process, the availability of new niches would, in turn, drive viral radiation, followed by further co-speciation. A similar evolutionary process was hypothesized for the related *Polyomaviridae* [52].

Therefore, strict co-speciation between papillomaviruses and their hosts does not explain the observed diversity. However, repeated events of niche sorting followed by virus-host-linked speciation were likely a vital determinant of the papillomavirus evolutionary history [48,53].

## 4 Minimal evidence of traditional Darwinian selection

Evolutionary selection is traditionally quantified by calculating the ratio of the number of non-synonymous substitutions per non-synonymous site to the number of synonymous substitutions per synonymous site (dN/dS). The dN/dS ratio is a useful measure of the strength and mode of natural selection acting on protein-coding genes. A dN/dS ratio close to 1 indicates neutral evolution. An excess of non-synonymous changes vs. synonymous changes (i.e., dN/dS > 1) argues that the codon (and the encoding amino acid) is under Darwinian or diversifying (positive) selection. Diversifying selection is often seen as the result of the genetic conflict. For example, in host-parasite interactions, the host and the parasite constantly adapt to each other. This "back-and-forth" evolution can result in rapid changes in both host and viral proteins, which can be detected as positive selection [54]. Negative selection (i.e., dN/dS < 1) is indicative of purifying selection. Under purifying selection, mutation of the codon and amino acid are believed to have a detrimental effect on the fitness of the organism [55].

Currently, there is limited evidence that papillomavirus genes or specific residues are evolving under positive selection [13,41]. An important caveat is that the use of highly divergent sequences saturates the evolutionary changes, thereby complicating the detection of selection signatures. However, even when comparing closely related viral genomes (i.e., variants sharing more than 90% nucleotide identity), only a few sites are under diversifying selection (reviewed in Ref. [13]). For example, analyses of HPV16 found only seven codons to be under diversifying selection [46,56,57]. Therefore, most papillomavirus genes are under strong purifying selection, thus restricting changes in the encoded proteins. Interestingly, most residues under diversifying selection are located within the E5, E6, and E7 proteins (reviewed in Ref. [13]). While the core – E1, E2, L2, and L1 – proteins may not tolerate mutations, the adaptive proteins – E5, E6, and E7 – appear more malleable [13,58,59]. This was demonstrated elegantly in a recent paper tracing the co-evolution between E6 and p53 [60]. The authors demonstrated a species barrier to p53 degradation by E6; human papillomavirus E6 proteins could degrade human p53 but not non-human primate p53. Vice-versa, macaque (MfPV10) E6 does not target human p53. This species specificity was mapped to a single amino acid in E6 and p53, demonstrating that E6 and p53 co-evolved in these viruses and their hosts [60].

## 5 Papillomaviruses use rare codons

The apparent lack of positive selection in papillomavirus genomes could suggest that the host is not exerting evolutionary selection on these viruses despite evolving alongside their hosts. However, evolutionary selection may act at the nucleotide level without changing the viral coding potential. Interestingly, it has been long known that papillomavirus’ codon usage is not matched to their hosts [61]. While the usage of these rare codons may seem sub-optimal from a host perspective, it seems likely that viruses benefit from this unmatched codon usage. We propose that the use of these rare codons limits the rate of synonymous and non-synonymous mutations (i.e., dN/dS) and thus, in part, restricts the ability to detect positive selection in papillomavirus genomes. Nonetheless, we will discuss data suggesting that much of the viral adaptation appears to happen at the nucleotide level without extensively changing the protein-coding potential.

### 5.1 Overlapping open reading frames and cis-acting binding sites restrict codon usage

Like many small DNA viruses, 85% of the papillomavirus genome encodes for viral proteins. In addition, the genome has many overlapping open reading frames. A codon has three nucleotide positions, allowing for three reading frames for each stretch of nucleotides. Therefore, one nucleotide change in an overlapping sequence could be synonymous in one ORF but is likely to be non-synonymous in an overlapping frame. This will significantly reduce the evolutionary space available to both ORFs. Regulatory motifs (e.g., transcription factor binding sites, *cis*-acting elements regulating splicing, etc.) are located within viral ORFs. The requirements imposed by these DNA elements reduce the available codon usage. Indeed, overlapping ORFs and *Cis*-acting binding sites further contribute to unusual codon usage patterns [62].

### 5.2 Risky codons restrict protein evolution

“Robust codons” code for amino acids with similar properties, while SNPs within “risky codons” are more ikely to result in nonsense mutations in coding sequences. For example, codons beginning with TpA are risky due to the chance of being mutated to a stop codon [63]. Interestingly, unlike human genes that almost exclusively use robust codons [64], papillomavirus genes are biased towards “risky codons” [65]. The preponderance of risky codons implies that many mutations will dramatically change the encoded amino acid and be detrimental to viral fitness and will therefore not become fixed in the viral population. This may partly explain the overwhelming purifying selection observed in papillomavirus genomes and maintain the balance between virus and host obtained through millions of years of linked evolution. Significantly, a rare non-detrimental mutation would dramatically alter the encoded amino acid and may expand the evolutionary space available to the thus mutated protein and allow for rapid expansion in a new niche.

## 6 Host-pathogen interactions shape the DNA sequence of papillomavirus genomes

As pointed out, few papillomavirus amino acids are under diversifying selection. This is somewhat surprising for viruses interacting with the host immune system [66]. However, typical methods (i.e., dN/dS-based approaches) designed to quantify evolutionary selection require amino acid changes. They do not directly quantify evolutionary selection acting at the level of the underlying nucleotide sequences.

**Fig. 2:**
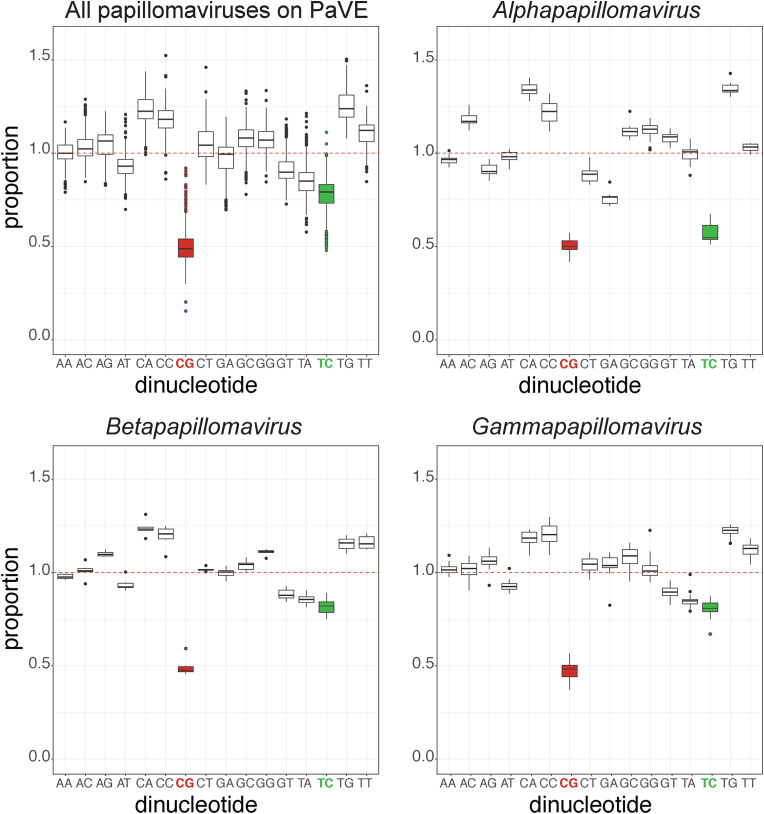
Dinucleotide frequencies across papillomavirus genomes. The observed-versus-expected (O/E) ratios of each dinucleotide in the papillomavirus genome sequences were calculated using a custom wrapper around the CompSeq program from the Emboss software suite. The red line at 1.0 indicates the ratio where a dinucleotide is seen as often as expected by chance. O/E ratios are displayed as box plots to show the minimum and maximum values (ends of the whiskers), interquartile range (length of the box), and median (line through the box). Outliers are shown as individual dots. CpG and TpC dinucleotides are highlighted in red and green, respectively. (A) All papillomaviruses in PaVE (n = 667). (B) Papillomaviruses are classified in the genus *Alphapapillomavirus* (n = 83). (C) Papillomaviruses classified in the genus *Betapapillomavirus* (n = 55). (D) Papillomaviruses classified in the genus *Gammapapillomavirus* (n = 100). A one-sample z-test was performed to obtain statistical support. In each panel, TpC and CpG were significantly less than 1 (p « 0.01). (For interpretation of the references to colour in this figure legend, the reader is referred to the Web version of this article.)

It has been long known that papillomavirus’ codons are different from their hosts. Indeed, by matching the codon usage of BPV L1 and L2 to codons more commonly used in humans, Zhou and colleagues significantly improved the expression of these proteins in human cells [61]. These data suggest that evolution may select nucleotide sequences and not only amino acid residues. By analyzing dinucleotide patterns, we and others identified that specific dinucleotide pairs are less common than what would be expected by chance [[67], [68], [69], [70]]. Specifically, there is a dramatic reduction in CpG and TpC dinucleotides (Fig. 2). The following paragraphs will discuss the potential implications of these reductions in genome content.

### 6.1 APOBEC3

APOBEC3 genes are a family of interferon-stimulated genes (ISGs). Thus, the activation of innate immune responses could lead to increased APOBEC3 activity [[71], [72], [73], [74], [75]]. This might especially be important for combating viruses acquired at mucosal sites of infection, where sentinel cell targets such as macrophages and dendritic cells are poised to respond to these antiviral responses. Indeed, ectopic expression of APOBEC3A restricts infection with HPV16 based pseudovirions [74], and induction of APOBEC3 expression leads to hypermutation of the HPV16 genome [76]. The human APOBEC3A, 3B, and 3H preferentially target TpC-containing sequences for cytidine deamination. APOBEC3 deamination of TpC residues can result in C-to-T transitions or C-to-G transversions, leading to hypermutation and inactivation of the viral genome [77]. Unlike many other viruses, papillomaviruses have not evolved to inhibit the activity of APOBEC3. Indeed, the expression of the viral oncogenes E6 and E7 may upregulate the steady-state levels of cellular APOBEC3 proteins [[77], [78], [79]].

**Fig. 3:**
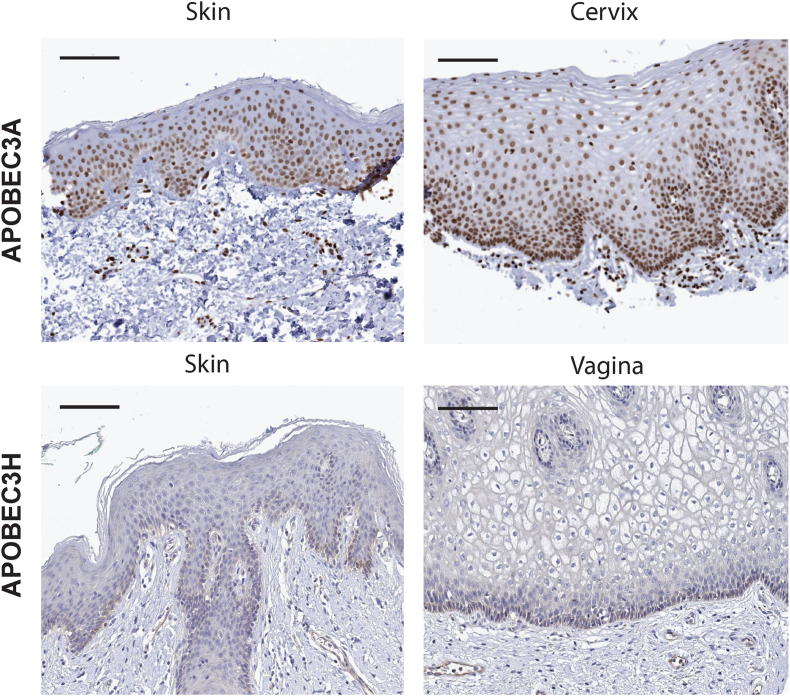
Expression level of APOBEC3 in human tissues. Images were obtained from The Human Protein Atlas, v21.proteinatlas.org. Scale bars represent 100 μm (A) APOBEC3A (Apolipoprotein B MRNA Editing Enzyme Catalytic Subunit 3A) stained epithelia. Normal skin tissue (T-01000, Male, age 36, Patient id: 1816) and normal cervical tissue (T-83000, Female, age 36, Patient id: 1773) were both stained with antibody HPA043237. (B) APOBEC3H (Apolipoprotein B MRNA Editing Enzyme Catalytic Subunit 3H) stained epithelia. Normal vulva tissue (T-80100, Female, age 66, Patient id: 3357) and normal vaginal tissue (T-81000, Female, age 34, Patient id: 1751) were both stained with antibody HPA021492.

APOBEC3-induced mutations would explain the observed reduction in TpC (Fig. 2, green symbols). Warren and colleagues used computer modeling to study this reduction in TpC [67]. The authors demonstrate that the decrease in TpC is mirrored by an increase in TpG and CpA dinucleotides, suggesting that APOBEC3-driven deamination is involved in these mutations. Importantly, APOBEC3A and APOBEC3H mRNA and protein levels are significantly higher in mucosal tissues compared with cutaneous skin ([67] and Fig. 3). Papillomaviruses infect mucosal tissues (i.e., a subset of *Alphapapillomaviruses)* and display the most dramatic reduction in TpC dinucleotide ratio. These data demonstrate a correlation between APOBEC3 expression and a decrease in TpC ratio.

Interestingly, viruses in the species *Gammapapillomavirus 6* infect the cervical niche and have a reduced TpC ratio similar to what is seen in mucosal *Alphapapillomaviruses* [67]. Based on the further evolutionary analysis, the authors propose that viral replication in tissues with high APOBEC3 expression resulted in hypermutation of the ancestral infecting virus. While most of these mutated viruses were less fit and were culled from the evolutionary tree, evolution selected ancestral papillomavirus genomes with reduced target sites in their genomes. Notably, the observed depletion in TpC is most pronounced when the cytidine is in the third codon position. Therefore, the cytidines in most of the remaining TpC dinucleotides seen in extant papillomavirus genomes are located in the first or second codon position. These observations suggest that the evolutionary loss of TpC dinucleotides likely did not change the encoded amino acids. Therefore, it seems that TpC reduction is limited by amino acid requirements, further highlighting that evolutionary selection occurs at the nucleotide sequence level with purifying selection maintaining amino acid composition and protein function. However, this also implies that APOBEC3 mutations in extant viral genomes will be non-synonymous and may impact viral fitness.

A recent paper sequenced the HPV16 genome from 5570 HPV16-infected case-control samples [80]. Because the authors used high-throughput sequencing, they could identify both major and minor allele frequencies for each sample. The authors identified rare mutations in the HPV16 genome consistent with APOBEC3 activity. These data imply that, following infection, APOBEC3 appears to further mutate a subset of the viral genomes in a cell. Interestingly, these APOBEC3 mutations were more common in HPV16(+) controls vs. HPV16(+) cervical precancer and cancer cases. The implications of these (APOBEC3 induced) mutations between cases and controls related to oncogenicity should be further examined. It will, however, be critical to account for the multiple bottlenecking events –infection, persistence, and transformation– during oncogenic progression.

Nonetheless, it is clear that APOBEC3 is mutating individual viral genomes during infection [80]. However, the evolutionary implications of this intra-patient variation are not clear. If evolutionary selection minimized APOBEC3 recognition sites within papillomavirus genomes, we could hypothesize that further reduction of TpC dinucleotides should reduce viral fitness, either in the patient or upon transmission. Under this hypothesis, minor variants with APOBEC3 mutation signatures should be rarely seen in consensus sequences available on GenBank. This is because the reduced fitness of the minor variants does not allow these variants to outcompete the major variant. While bulk sequencing and reporting the consensus sequence, these minor variants are effectively ignored. To test the hypothesis that APOBEC3 mutations are rare in consensus extant sequences, we analyzed the genomes of ∼595 complete HPV16 genomes available on GenBank (Table 1). The data suggests that APOBEC3 mediated mutations of TpC sites are indeed rare in bulk, normalized sequences. In the 595 genomes, we identified 33 unique APOBEC3 mutations.

**Table 1:** APOBEC3 mutations in extant HPV16 variant genomes.

**gene (HPV16)**	**consensus**	**mutant**	**consensus**	**mutant**	**count**	**effect of mutation**
E1 (n = 595)	CTA	TTA	L	L	1	synonymous
E1 (n = 595)	CAG	GAG	Q	E	3	non-synonymous
E1 (n = 595)	TCA	TTA	S	L	1	non-synonymous
E1 (n = 595)	CTA	TTA	L	L	114	synonymous
E1 (n = 595)	TCT	TTT	S	F	1	non-synonymous
E1 (n = 595)	TCC	TTC	S	F	1	non-synonymous
E1 (n = 595)	TCT	TTT	S	F	1	non-synonymous
E2 (n = 596)	CTT	TTT	L	F	1	non-synonymous
E2 (n = 596)	TCT	TTT	S	F	2	non-synonymous
E2 (n = 596)	CCT	GCT	P	A	116	non-synonymous
E2 (n = 596)	ATC	ATT	I	I	2	synonymous
E2 (n = 596)	CCA	TCA	P	S	10	non-synonymous
E6 (n = 596)	CAG	GAG	Q	E	1	non-synonymous
E6 (n = 596)	CCA	TCA	P	S	1	non-synonymous
E6 (n = 596)	CGG	TGG	R	W	1	non-synonymous
E6 (n = 596)	TCT	TGT	S	C	1	non-synonymous
E7 (n = 598)	CTC	CTT	L	L	1	synonymous
E7 (n = 598)	CGG	TGG	R	W	1	non-synonymous
L1 (n = 596)	GTC	GTT	V	V	1	synonymous
L1 (n = 596)	TCA	TTA	S	L	1	non-synonymous
L1 (n = 596)	CCA	GCA	P	A	1	non-synonymous
L1 (n = 596)	TCA	TTA	S	L	1	non-synonymous
L1 (n = 596)	CTA	TTA	L	L	116	synonymous
L1 (n = 596)	TCC	TTC	S	F	1	non-synonymous
L2 (n = 596)	CCC	TCC	P	S	1	non-synonymous
L2 (n = 596)#	CCA	GCA	P	A	1	non-synonymous
L2 (n = 596)#	CCA	TCA	P	S	1	non-synonymous
L2 (n = 596)	TCT	TTT	S	F	1	non-synonymous
L2 (n = 596)$	TTC	TTG	F	L	46	non-synonymous
L2 (n = 596)$	TTC	TTT	F	F	194	synonymous
L2 (n = 596)	TCA	TTA	S	L	4	non-synonymous
L2 (n = 596)	TCA	TTA	S	L	2	non-synonymous
L2 (n = 596)	CCT	TCT	P	S	1	non-synonymous

HPV16 sequences were downloaded from GenBank using the PaVE database. Individual HPV16 genes were aligned, and a consensus genome was calculated. The associated amino acid was identified for each SNP that changes TpC in the consensus to TpT or TpG. The occurrence of each SNP in different genomes (count) and the effect of the mutation are indicated. # and $ indicate SNPs at the same position in L2 that were mutated in at least two separate events.

Furthermore, only seven of these mutations were synonymous. While some of these mutations were observed in ∼20% of the samples, most mutations were only observed in a single sequence. Overall, the synonymous mutations were identified in more individual samples. An interesting example is a mutation in L2 (indicated with $). This TpC dinucleotide was mutated in at least two separate instances (TTC→TTG and TTC→TTT). While the TTT mutation is silent, the TTG mutation changes the wildtype F to a variant L at this position. In line with the hypothesis that most extant TpC mutations will affect viral fitness, the silent mutation is seen in 4 times more samples than the non-synonymous mutation. Therefore, it appears that while intra-patient APOBEC3 editing alters a population of the viral genomes, the global effects of APOBEC3 mutations on HPV16 evolution may be more limited. Potentially, the bottleneck of spreading from one patient limits the evolutionary landscape available to HPV16 and other papillomavirus genomes.

The analysis in Table 1 also demonstrates that APOBEC3 is indeed editing HPV genomes and that the increased TpG proportions (Fig. 2; *Alphapapillomavirus*) are not just a mathematical effect of maintaining overall T content in the face of lowering TpC proportions.

### 6.2 TLR9

**Fig. 4:**
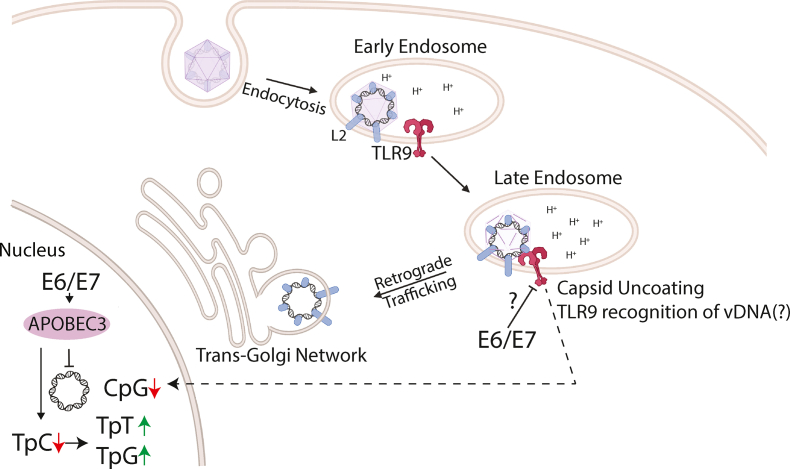
Model of infection highlighting APOBEC3 and TLR9. The papillomavirus lifecycle risks detection by TLR9 as well as antiviral activities mediated by APOBEC3. Internalized virions enter the endolysosomal pathway triggering pH-dependent uncoating. This uncoating likely exposes the CpG dinucleotides in viral DNA to endosomal TLR9. However, evolution selected for viral genomes with depleted CpG reducing the ability of TLR9 to detect the viral DNA during entry. Interestingly, viral oncogenes have been shown to degrade TLR9 suggesting that the viral lifecycle benefits from degrading TLR9 receptors. The viral L2 protein directs trafficking to the trans-Golgi network. The viral E6/E7 proteins induce the steady-state levels of APOBEC3, which could hypermutate the viral genome leading to loss of persistent infection. However, the viral genomes evolved a reduced TpC content, thus evading spurious mutations by APOBEC3.

Papillomaviruses access mitotically active basal cells through lesions in the stratified epithelia of cutaneous or mucosal tissues. The virus is endocytosed by binding to cellular receptors, and the viral capsid disassembles, exposing the viral genome (Fig. 4) [[81], [82], [83], [84], [85], [86], [87]]. The exposed viral DNA may be recognized by endosomal TLR9, resulting in a downstream inflammatory immune response [[88], [89], [90], [91], [92]].

As shown in Fig. 2, in addition to a reduction in TpC, papillomavirus genomes substantially reduce CpG dinucleotides. This is in part due to the mutagenic effects of 5-methylcytosines. CpGs are frequently methylated, and the resulting 5-methylcytosines are prone to deamination leading to C→T mutations. It is likely that part of the observed CpG depletion in papillomavirus genomes can be explained by this phenomenon [13,69,70]. However, the deficit in CpG is not mirrored by an expected increase in TpG (Fig. 2). Therefore, King and colleagues tested the hypothesis that similar to TpC depletion, this CpG depletion results from an evolutionary response to antiviral effectors. King and colleagues analyzed papillomavirus genomes isolated from bats. It was previously shown that bat TLR9 is under diversifying selection in bats [68,93]. Given the co-evolution theory between PVs and their hosts, this change in TLR9's DNA recognition should induce a complementary response in the viral genome. Since the DNA recognition domain of bat TLR9 is under evolutionary selection, the authors compared papillomavirus genomes isolated from different bat species belonging to different suborders. The authors demonstrate that viruses infecting specific bats reduce their genomic CpG within a known TLR9 recognition motif (‘ACGT’). Furthermore, the authors demonstrate that members of the genus *Alphapapillomavirus* significantly deplete TpC in the context of TCGA motifs, a known human TLR9 recognition motif [94,95,[95], [95], [96], [97]].

Together, these data demonstrate that papillomaviruses deplete CpG in the context of species-specific TLR9 recognition motifs. Since the authors detected evidence of co-evolution between TLR9 and the viral nucleotide ratio, these changes are likely due to evolutionary pressure provided by evading recognition by the TLR9 protein. Similar to what was seen as a response to APOBEC3 editing, the depletion of CpG does not appear to drastically alter the amino acid content of the viral proteins [68]. These data support the idea that papillomavirus protein sequences are evolutionarily constrained but that nucleotide sequences are evolving to escape antiviral responses shaping host-virus interactions.

## 7 Role of immune effectors during persistent infections

Overall, the reduction in TpC ratio suggests that papillomaviruses evolved to avoid APOBEC3-mediated editing of their genomes. Yet, papillomavirus infections upregulate the steady-state levels of APOBEC3 in infected cells. This dichotomy was recently addressed in a thoughtful review by Wallace and Munger [98]. It is well known that the viral E7 proteins, which infect the mucosal tissues, degrade pRb. pRb has been demonstrated to play critical roles in silencing repetitive elements. Therefore, degradation of pRb by E7 is expected to increase the transcription of repetitive elements [99]. Expression of Long INterspersed Element-1 (LINE1 or L1) and other repetitive elements would generate neoantigens, potentially activating the adaptive immune response. The LINE1 ORF 2 is an endonuclease that, while essential to retrotransposition, may cause excessive double-strand DNA breaks leading to senescence or apoptosis. Activation of the adaptive immune system and/or apoptosis would lead to eliminating HR-HPV–infected cells. APOBEC3s are known to restrict the expression of LINE1 and other repetitive elements [100]. Therefore, increased expression of APOBEC3 may be beneficial for HPVs, and secondary viral genome editing is minimized by reducing the available TpC sites in the genome.

Similarly, the reduction in CpG in the context of a TLR9 recognition motif suggests that TLR9 senses HPVs during infection. However, unlike APOBEC3, the viral oncogenes may reduce TLR9 levels in infected cells [[101], [102], [103], [104]]. However, E6 and E7 are only expressed after the viral genome traffics to the nucleus, presumably after endosomal sensing of the viral DNA has occurred. This suggests that interfering with TLR9 activity may benefit a papillomavirus’ ability to persist long-term in cells. Indeed, clearance of MmuPV1 infection in the mouse papillomavirus model (MmuPV1) is, in part, regulated by a functional MyD88 signaling pathway, suggesting a role for TLRs in the immune response against established papillomavirus infections [105]. Interestingly, other DNA viruses (e.g., Merkel cell polyomavirus, hepatitis B virus, and Epstein-Barr virus [EBV]) also interfere with TLR9 function during the maintenance phase of the infection [[106], [107], [108]]. This raises an interesting hypothesis that, in addition to evading TLR9 during infectious entry (by reducing the genomic CpG content), the virus benefits from interfering with the TLR9 pathway (following expression of E6 and E7).

## 8 Implications and future work

In the above sections, we discussed current data suggesting that evolutionary processes shape the nucleotide sequences of papillomavirus genomes despite robust purifying selection occurring at the amino-acid/protein level. These observations likely have important implications for how we think about viral evolution and study the papillomavirus lifecycle.

### 8.1 Studying papillomavirus evolution

Papillomaviruses infect fish, relying on the theory of co-speciation, this implies that the ancestral papillomavirus was already infecting animals in the Palaeozoic (lasting from 540 to 250 million years ago) when fish evolved. Indeed, these dates are supported by evolutionary analyses dating the most recent common ancestor of all papillomaviruses to roughly 450 million years ago [58,109]. Despite the observation that papillomaviruses evolve slowly (roughly 5x faster than the infected host; [13]), over this timescale, much of the DNA sequence information has become saturated. Briefly, genetic saturation occurs when multiple substitutions happen at the same site in a sequence such that the apparent sequence divergence rate is lower than what has happened. The longer the time frame, the more likely this is an issue. Practically, amino acid residues are less sensitive to saturation, allowing for studying more ancient evolutionary events [110]. Indeed, the phylogenetic tree in Fig. 1A was constructed using protein sequences. However, this review argues that much of the evolutionary information is contained within the nucleotide sequences, which is lost when solely considering the encoded amino acids. A recent study by the Chen and Burk labs used trimer spectra to construct a dendogram of the *papillomaviridae* [70]. This alignment-free approach relies on the distribution of trimers of DNA sequences and using this information to cluster similar sequences into a UPGMA-based dendrogram [[111], [112], [113], [114], [115], [116]].

Interestingly, this approach clusters the mucosal members of the genus *Gammapapillomavirus* with the mucosal viruses belonging to the genus *Alpapapillomavirus*. Strikingly, these viruses show a similar tissue tropism and have undergone similar TpC reduction due to interactions with APOBEC3 in the mucosal tissue (see above). These relationships are not apparent using more conventional homology-based analyses. Therefore, combinations of conventional homology-based and these k-mer-based approaches may be needed to dissect the evolutionary history of the *Papillomaviridae* fully. However, it is clear that traditional dN/dS-based methods have significantly underestimated the evolutionary processes involved in shaping papillomavirus diversity and fitness.

### 8.2 Importance of nucleotide motif depletion

This review highlighted the importance of TpC and CpG depletion. However, Fig. 2 highlights that other dinucleotides are depleted from viral genomes. Furthermore, as illustrated by APOBEC3 in mucosal tissues, this depletion may be more apparent when considering specific clades of the phylogenetic tree or even host environmental niches. For example, the *Betapapillomavirus* and *Gammapapillomavirus* graphs in Fig. 2 show a reduction in TpA. This may be partly because the universal stop codons contain TpA. However, if this were the case, we would expect a similar decrease in TpA across all papillomaviruses. Since this is not the case, it would be interesting to explore whether the reduction in TpA results from co-evolution with antiviral responses.

Interestingly, a depletion in TpA has been linked to the observation that, when transcribed, UpA is the preferred target for ribonucleases. This was supported by the observation that TpA depletion is most robust in exons of protein-coding genes [117]. However, a computational study also suggests that the apparent TpA depletion is due to a mathematical artifact and is directly linked to the decrease in CpG [118]. Beyond dinucleotides, it is likely informative to study extended motifs.

### 8.3 Effects of silent mutagenesis approaches

Studying the function of viral proteins often involves mutagenesis of a specific residue within this viral protein. Since many papillomavirus genes overlap other genes, most researchers will ensure that the generated mutation does not alter the protein encoded in the next overlapping frame. However, accumulating data suggest that the nucleotide sequence, and not just the encoded protein(s), may substantially impact viral function and fitness. The data summarized in this review suggests that these mutational studies should be carefully designed and interpreted.

### 8.4 Intrapatient mutations and cancer progression

A recent paper by Mirabello and colleagues demonstrates a correlation between specific HPV16 SNPs and worse survival in human papillomavirus-driven oropharyngeal cancers [119]. The same authors demonstrate that APOBEC3-induced mutations in the HPV16 genome are associated with viral clearance [120]. Together with other reports, these data demonstrate that at least a subset of papillomavirus genomes acquire mutations during infection and cancer progression. The data presented in this review argues that these intrapatient mutations are not a major contributor to the current papillomavirus diversity (Table 1). Indeed, HPV16 variant lineages likely diverged between 400 and 600 thousand years ago, predating the divergence of modern *Homo sapiens* [121,122]. Importantly, papillomavirus fitness is measured by a virus' ability to persist long-term in a host while maximizing transmission to the next host. Therefore, these processes regulate the balance between nucleotide level evolution as described in this review and purifying selection at the protein level. However, the evolutionary pressures are likely very different within a patient and certainly during progression towards cancer. As such, these cancer specific SNPs may be causative of cancer progression. Therefore, studying the impact of intrapatient mutation, selection, and evolution will be necessary. High-throughput sequencing has made these types of experiments feasible [80,[123], [124], [125]]. By controlling for the many bottlenecks during cancer progression, researchers may be able to identify mutations within viral genomes associated with cancer, allowing for more personalized screening of patients.

### 8.5 Impact of antiviral restriction factors during persistent infection

As discussed above, papillomavirus genomes are depleted in CpG to evade detection by TLR9 [68]. However, the viral oncogenes regulate the steady-state levels of this protein in infected cells [[101], [102], [103], [104]]. This suggests that, during persistent infection, when the viral DNA is mainly located in the nucleus, the virus benefits from interfering with the TLR9/MyD88/IFN pathway [126]. This is reminiscent of papillomavirus interactions with another arm of the innate immune system, cGAS/STING. cGAS recognizes cytoplasmic DNA and activates STING resulting in the activation of the interferon response. However, it was recently demonstrated that the papillomavirus genome is not sensed by cGAS during infection [126]. Yet, the papillomavirus E6 and E7 proteins have been implicated in interfering with the cGAS/STING pathway [[127], [128], [129], [130], [131]]. Together, these data suggest that papillomaviruses interfere with antiviral pathways to promote long-term infections, not just acute infection.

If papillomaviruses indeed interfere with TLR9 activity to promote long-term infections, this raises an important question about what PAMP (pathogen-associated molecular pattern) or DAMP (damage-associated molecular patterns) the cellular TLR9 may be detecting. Since papillomaviruses replicate in the nucleus of the infected cell, it is unlikely that TLR9 is detecting the viral DNA. Moreover, since the depletion of CpG dinucleotides likely further reduces TLR9 recognition, it is doubtful that the viral genome represents PAMP recognized by TLR9. This raises the possibility that persistent papillomavirus infections induce the production of DAMPs and that papillomaviruses evolved to evade the innate immune system in two complementary ways, first by escaping detection during infectious entry (e.g., by editing the genome content) into the cell and second by interfering with the pattern recognition receptors following infection.

## 9 Conclusions

This review discusses current evidence that papillomaviruses are evolving in response to interactions with the innate immune system. Interestingly, there is limited evidence of adaptive evolution in papillomavirus proteins. However, we propose that papillomavirus evolution primarily occurs at the nucleotide level in a way that optimizes viral fitness without altering the encoded aminoacids and thus remaining constrained by pervasive purifying selection at the protein level.

## Author statement

KMK: Figure preparation and Text writing/editing EVR: Figure preparation and Text writing/editing IGT: Figure preparation and Text writing/editing KVD: Figure preparation and Text writing/editing.

## Declaration of competing interest

The authors declare that they have no known competing financial interests or personal relationships that could have appeared to influence the work reported in this paper.

## Data Availability

All sequence data is available from public databases
